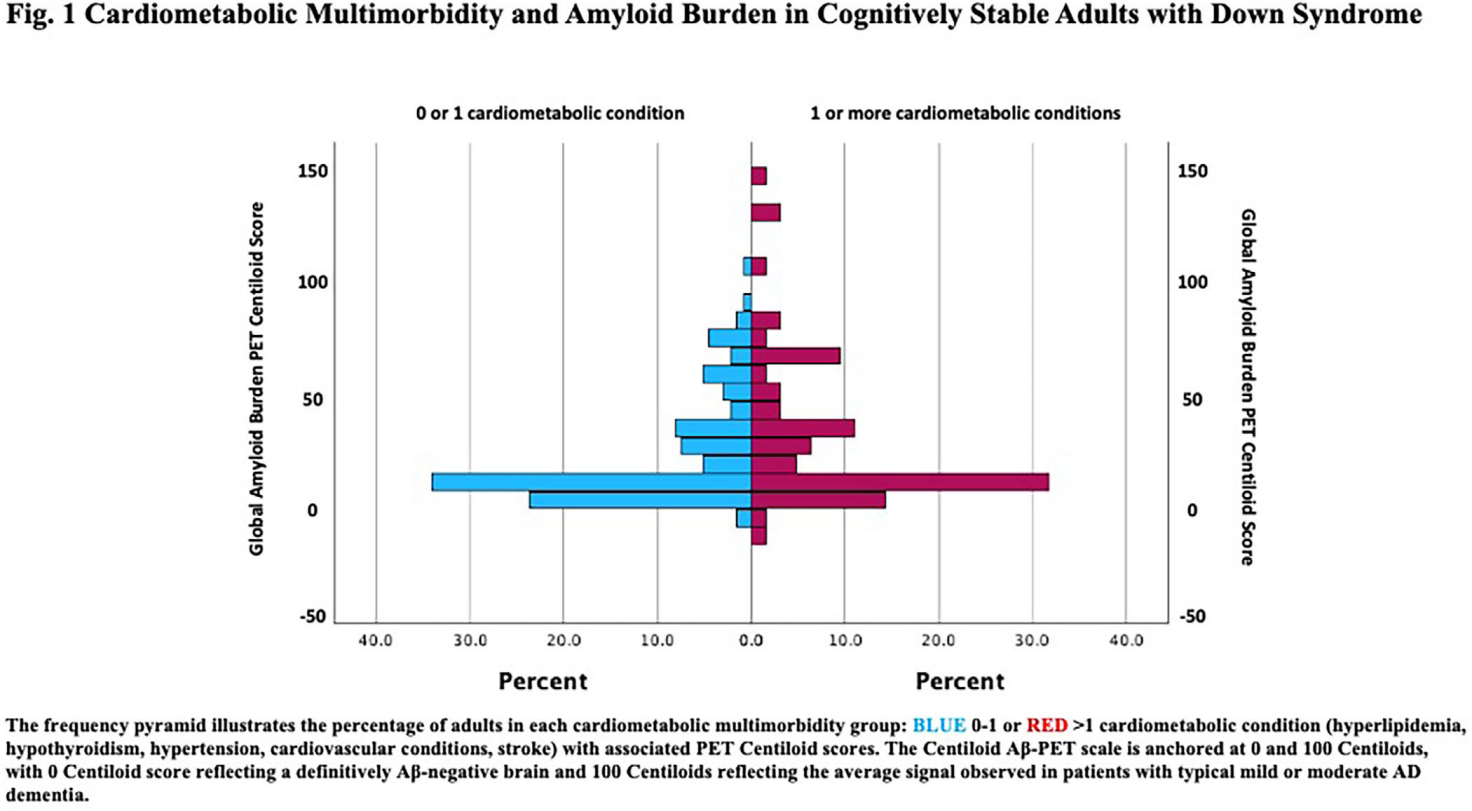# Cardiometabolic multimorbidity is associated with greater amyloid burden in cognitively stable adults with Down Syndrome

**DOI:** 10.1002/alz.095368

**Published:** 2025-01-09

**Authors:** Stephanie Tuxen Bisgaard, Jennifer Bruno

**Affiliations:** ^1^ Stanford University, Stanford, CA USA; ^2^ Stanford, Palo Alto, CA USA

## Abstract

**Background:**

Nearly everyone (>90%) with Down Syndrome (DS) develops Alzheimer’s Disease (AD), and neuropathological development is studied extensively in this group. However, there is a gap in our understanding of AD age onset variation in DS, from 40 to 70 years. Cardiometabolic conditions (CC) are known dementia risk‐factors in the general population, and cohort‐studies document that cardiometabolic multimorbidity (CM) synergistically increases MCI, dementia, and AD. CC are highly prevalent in DS. Meanwhile, amyloid burden is associated with AD and elevated in DS across lifespan. However, there are no cohort studies on the association between CM and amyloid burden in DS. We tested the association between CM and amyloid burden in DS.

**Method:**

We studied baseline data from 240 participants, 25‐65 years (198 cognitively stable and 42 with MCI or dementia), from the Alzheimer’s Biomarker Consortium‐Down Syndrome (ABC‐DS) longitudinal study. Participants were grouped in 0‐1 or >1 CC (hyperlipidemia, hypothyroidism, hypertension, cardiovascular conditions, stroke). Participants completed an amyloid positron emission tomography (PET) scan with [¹¹C]‐Pittsburgh compound B ([¹¹C]‐PiB) or [¹⁸F]‐AV45 (florbetapir). Regional standard uptake value ratios (SUVRs) were calculated relative to the cerebellar cortex and transformed to Centiloid global amyloid burden scores for further analysis. We tested the association between CM and amyloid burden scores using hierarchical linear regression, including age and apolipoprotein E (ApoE) status as covariates.

**Result:**

The regression model revealed that CM was significantly associated with greater amyloid burden after accounting for age and ApoE status [F (3,194) = 57.752, *p*<0.001, ß = 0.228, 95% CI [0.007 ‐ 0.449], *p* = 0.043]. Centiloid values were higher for those with two or more CC (Figure 1). We did not find a significant association among participants with either MCI or dementia, possibly due to small samples within these categories.

**Conclusion:**

Our results suggest that incidence of two or more CC synergistically affect amyloid burden in cognitively stable adults with DS. These findings can help us understand the prodromal role of CM in accelerating AD in DS and provide insights to preventative strategies of relevance to the general population. We plan future studies examining CM and related biomarkers, eg., chronic peripheral inflammation, influence AD in DS.